# *Ngly1* ^−/−^ rats develop neurodegenerative phenotypes and pathological abnormalities in their peripheral and central nervous systems

**DOI:** 10.1093/hmg/ddaa059

**Published:** 2020-04-07

**Authors:** Makoto Asahina, Reiko Fujinawa, Sayuri Nakamura, Kotaro Yokoyama, Ryuichi Tozawa, Tadashi Suzuki

**Affiliations:** 1 Takeda-CiRA Joint Program (T-CiRA), Kanagawa 2518555, Japan; 2 T-CiRA Discovery, Takeda Pharmaceutical Company Ltd., Kanagawa 2518555, Japan; 3 Glycometabolic Biochemistry Laboratory, RIKEN Cluster for Pioneering Research, Saitama 3510198, Japan; 4 Nonclinical Safety Research, Axcelead Drug Discovery Partners Inc., Kanagawa 2510012, Japan

## Abstract

N-glycanase 1 (NGLY1) deficiency, an autosomal recessive disease caused by mutations in the *NGLY1* gene, is characterized by developmental delay, hypolacrima or alacrima, seizure, intellectual disability, movement disorders and other neurological phenotypes. Because of few animal models that recapitulate these clinical signatures, the mechanisms of the onset of the disease and its progression are poorly understood, and the development of therapies is hindered. In this study, we generated the systemic *Ngly1*-deficient rodent model, *Ngly1*^−/−^ rats, which showed developmental delay, movement disorder, somatosensory impairment and scoliosis. These phenotypes in *Ngly1*^−/−^ rats are consistent with symptoms in human patients. In accordance with the pivotal role played by NGLY1 in endoplasmic reticulum-associated degradation processes, cleaving N-glycans from misfolded glycoproteins in the cytosol before they can be degraded by the proteasome, loss of *Ngly1* led to accumulation of cytoplasmic ubiquitinated proteins, a marker of misfolded proteins in the neurons of the central nervous system of *Ngly1*^−/−^ rats. Histological analysis identified prominent pathological abnormalities, including necrotic lesions, mineralization, intra- and extracellular eosinophilic bodies, astrogliosis, microgliosis and significant loss of mature neurons in the thalamic lateral and the medial parts of the ventral posterior nucleus and ventral lateral nucleus of *Ngly1*^−/−^ rats. Axonal degradation in the sciatic nerves was also observed, as in human subjects. *Ngly1*^−/−^ rats, which mimic the symptoms of human patients, will be a useful animal model for preclinical testing of therapeutic options and understanding the detailed mechanisms of NGLY1 deficiency.

## Introduction

N-glycanase 1 (NGLY1), also known as peptide:*N*-glycanase, is an evolutionarily conserved enzyme among eukaryotes (1,2). NGLY1 plays a crucial role in quality control for newly synthesized N-glycoproteins. During the endoplasmic reticulum-associated degradation (ERAD) process, misfolded N-glycoproteins are retrotranslocated from the endoplasmic reticulum (ER) lumen to the cytosol. NGLY1 cleaves N-glycans from misfolded glycoproteins in the cytosol while they are degraded by the proteasome (1–5).

Mutations in the *NGLY1* gene are associated with a rare congenital disorder affecting young children (6–9). Almost all *NGLY1* missense and nonsense mutations reduce NGLY1 protein levels and enzymatic activity (10). In 2012, the first patient harboring mutations in *NGLY1* was identified (11), and more than 50 patients have been confirmed worldwide (Matt Wilsey, Grace Science Foundation, personal communication). Several clinical papers on NGLY1-deficient patients have been published and report that the typical clinical features of NGLY1 deficiency include developmental delay, hypolacrima or alacrima, seizure, intellectual disability, motor deficit and movement disorders that can include chorea, athetosis, dystonia, myoclonia, tremor and dysmetria (6–9,12,13). Mutants of *Ngly1* and its orthologs have been analyzed in various organisms to elucidate the molecular function of NGLY1 (Supplementary Material, Table S1) (1,14–26). However, the pathogenesis of NGLY1 deficiency is poorly understood, and no effective therapy is currently available.

The creation of systemic *Ngly1*-deficient rodent models that mimic the clinical features of NGLY1-deficient patients is essential for elucidating the mechanisms of NGLY1 deficiency and for evaluating therapeutic options in preclinical studies. A previous study reported that homozygous *Ngly1* knockout mice derived from C57BL/6 showed embryonic lethality, whereas the heterozygous mice were fertile and did not show any obviously recognizable phenotypes (26). Although useful for rescuing the embryonic lethality and elucidating mechanisms of neurological. dysfunction, conditional knockout rodents have limited value because they do not recapitulate the systemic loss of target genes (27). Fujihira et al. reported that a C57BL/6- and ICR-mixed genetic background could rescue the embryonic lethality in *Ngly1*-deficient mice, indicating the importance of genetic background in the embryonic lethality of *Ngly1* deficiency (26).

The rat is recognized as a rodent more suitable for neurobehavioral studies than the mouse (28). The larger body size of rats may allow us to obtain large enough biological samples of blood, urine, cerebrospinal fluid and tissues to elucidate neurobehavioral phenotypes in *in vivo* models. Most behavioral phenotyping tasks have been developed in rats and are popular, and most behavioral studies have been carried out in outbred rat strains, such as Wistar, Sprague–Dawley (SD) and Long–Evans rats (29). Furthermore, rats are suited to water maze tests to study learning and memory (30), and they display sophisticated social and cognitive behaviors (31).

In this study, we established a novel animal model of NGLY1 deficiency using SD rats. We examined their phenotypes and confirmed that *Ngly1*^−/−^ rats develop neurological symptoms similar to NGLY1-deficient human subjects. We also explored pathological abnormalities potentially associated with the neurodegenerative phenotypes in *Ngly1*^−/−^ rats to elucidate the pathological mechanisms of NGLY1 deficiency. This rat could serve as a new model animal for conducting preclinical studies for NGLY1 deficiency as well as providing insights into the pathophysiology of this devastating genetic disorder.

## Results

Here we show data on male rats. Data on the phenotypes of female rats are described in Supplementary Material, Figs S1 and S2.

### Generation, appearance and body weights of *Ngly1*^−/−^ rats

**Figure 1:**
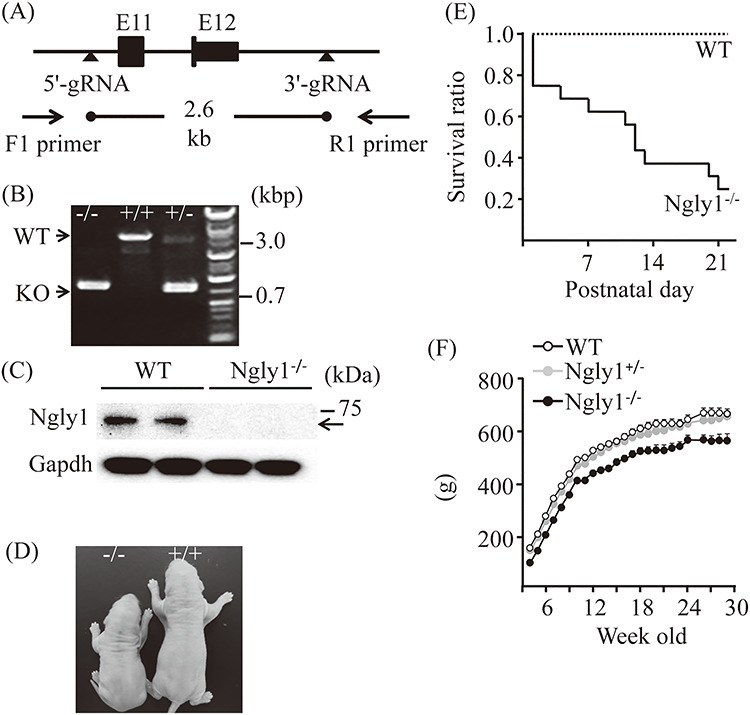
Generation, appearance and body weights of *Ngly1*^−/−^ rats. (**A**) Schematic figure showing generation of *Ngly1*^−/−^ rat using CRISPR/Cas9 genome editing technology. (**B**) Representative results from genotyping of *Ngly1*^+/+^, ^+/−^ and ^−/−^ rats. Deletion in the *Ngly1* gene can be detected by PCR and electrophoresis. (**C**) Western blot analysis of NGLY1 expression in brains from *Ngly1*^−/−^ and WT rats. (**D**) Representative photograph of *Ngly1*^−/−^ rat and a littermate WT rat at postnatal day one. (**E**) Survival curve of WT and *Ngly1*^−/−^ rats (WT, *n* = 10; KO, *n* = 20). *Ngly1*^−/−^ rats have a significantly shorter lifespan than their littermates, WT rats, until weaning. (**F**) Body weight of *Ngly1*^−/−^, *Ngly1*^+/−^ and WT rats (*Ngly1*^−/−^; *n* = 10–12, *Ngly1*^+/−^; *n* = 12, WT; *n* = 12). Rats were weighed weekly after weaning (starting from 4 weeks old).

To generate *Ngly1*-deficient rats, we employed genome editing technology. The *Ngly1*^−/−^ rat has approximately 2.6 kb deletions in exon 11 and exon 12 and a 3′ flanking region of the *Ngly1* gene (Fig. 1A). Genotyping by polymerase chain reaction (PCR) identified wild-type (WT) (+/+), heterozygous (+/−) and homozygous alleles (−/−) (Fig. 1B). There was no detectable NGLY1 protein in the whole brain lysate of *Ngly1*^−/−^ rats (Fig. 1C). When we carried out heterozygote mating, *Ngly1*1^−/−^ rats were born in a manner slightly deviated from the Mendelian ratio (WT/heterozygous/homozygous = 0.6:1.0:0.4). When *Ngly1*^−/−^ rats were reciprocally bred with *Ngly1*^−/+^ or *Ngly1*^−/−^ rats, no female *Ngly1*^−/+^ or *Ngly1*^−/−^ rat became pregnant, and there was no pup in these mating patterns, suggesting that *Ngly1*^−/−^ causes infertility in rats. At birth, *Ngly1*^−/−^ rats were smaller than their littermates (body weight: male WT 7.8 ± 0.23 g (*n* = 10), male *Ngly1*^−/−^, 5.2 ± 0.14 g (n = 26), mean ± standard error of the mean [SEM]) and distinguishable visibly from WT littermates (Fig. 1D). *Ngly1*^−/−^ rats have a reduced lifespan, and 70% of *Ngly1*^−/−^ rats died before weaning, whereas littermate WT and *Ngly1*^−/+^ rats did not (Fig. 1E). However, once the rats escape the early developmental death before weaning, 10 out of 12 male *Ngly1*^−/−^ rats survived 6 months. The mean body weight of male *Ngly1*^−/−^ rats was significantly lower than that of WT and *Ngly1*^−/+^ rats (Fig. 1F), although there was a sex difference in the weight change of *Ngly1*^−/−^ rats. Both male and female *Ngly1*^−/−^ rats showed significantly reduced body weights compared with WT rats until they were 7 weeks of age. However, female *Ngly1*^−/−^ rats gradually gained weight and showed increased body weight after 13 weeks of age, compared with WT and *Ngly1*^−/+^ rats (Supplementary Material, Fig. S1A). The brain weights of *Ngly1*^−/−^ rats were significantly lower than those of WT rats at 5, 15 and 29 weeks of age (Supplementary Material, Fig. S2), implying microcephaly in *Ngly1*^−/−^ rats, as observed with NGLY1-deficient patients (6–9,12).

Tail necrosis is one of the most prominent visible phenotypes of *Ngly1*^−/−^ rats (Supplementary Material, Fig. S3A and B). The penetrance of this phenotype was high (male, 11/12 rats; female, 16/16 rats at 29 weeks of age). Scoliosis, which is one of the characteristics of NGLY1 deficiency subjects (6–9,13), was also observed in older *Ngly1*^−/−^ rats at 29 weeks of age (Supplementary Material, Fig. S3C and D), but not at 5 and 15 weeks of age, although the penetrance was not high, and there was a sex difference in the penetrance (male, 6/10 rats; female, 2/10 rats).

### *Ngly1* ^−/−^ rats have somatosensory impairment

In the pain reflex tests of the functional observational battery (FOB), while all WT rats quickly moved forward to escape from the stimulus or bit at it immediately after looking back, some of the *Ngly1*^−/−^ rats slowly looked back or moved forward to escape from the stimulus, indicating an impaired pain reflex. In the FOB auditory reflex tests, some *Ngly1*^−/−^ rats did not show any reaction, but WT rats hesitated at the stimulus or moved their ears as an auditory reflex. Incidence of somatosensory impairment in WT rat is 0 out of 10 rats in both pain reflex and auditory reflex, while in Ngly1^−/−^ rats, 3 out of 10 rats showed impaired pain reflex and 4 out of 10 rats showed impaired auditory reflex. These results suggest that *Ngly1*^−/−^ rats have somatosensory impairment with reduced auditory and pain reflexes.

### *Ngly1* ^−/−^ rats developed motor deficit

**Figure 2:**
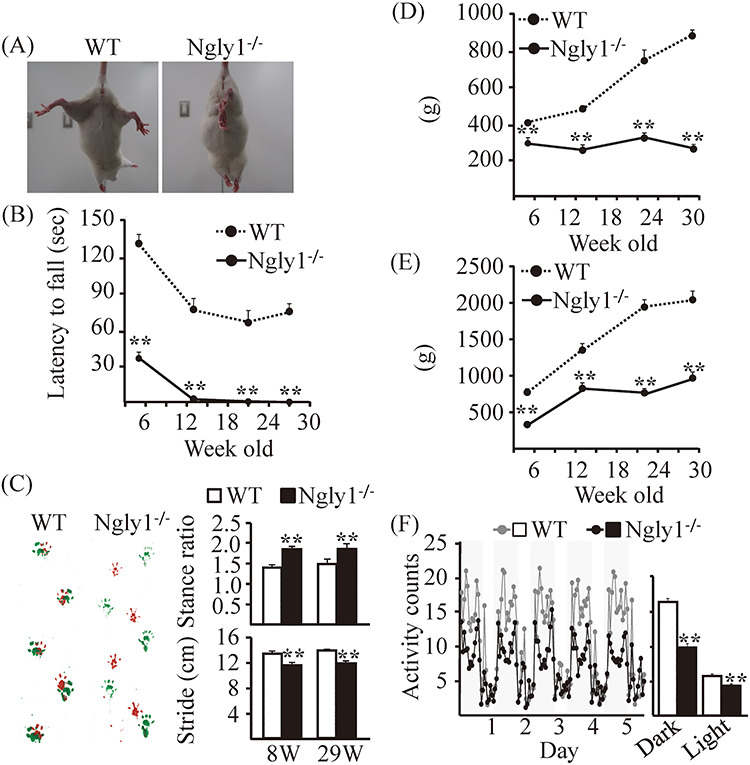
Motor dysfunction in *Ngly1*^−/−^ rats. (**A**) Abnormal hindlimb clasping of *Ngly1*^−/−^ rats compared with WT rats when suspended by the tail. (**B**) Rotarod testing for motor coordination of *Ngly1*^−/−^ and WT rats at several ages. The time until drop from the accelerating rod (4–40 rpm in 4 min) is shown. (**C**) Gait analysis: left, representative paw placement records of 29-week-old rats; right, stride lengths and stance ratios of rats. (**D, E**) Grip-strength tests for assessment of forelimb (D) or forelimb and hindlimb (E) muscle force. (**F**) Home cage activity recorded over 120 h (left). The graph represents the means of 5-day averages of each analysis period (right). Values represent means ± SEM. The number of rats examined is 10–12 each. Asterisks indicate ^*^^*^P < 0.01, ^*^P < 0.05.

Apparent movement deficits in NGLY1-deficient subjects are observed in their early childhood (6–9). We compared motor behavior between WT and *Ngly1*^−/−^ rats at 5, 15 and 29 weeks of age. *Hindlimb clasping*: *Ngly1*^−/−^ rats developed an abnormal hindlimb clasping reflex after 4 weeks of age when they were suspended by their tails, whereas WT rats show a characteristic response, trying to escape by splaying their hind limbs away from the trunk of their body (Fig. 2A, Supplementary Material, Videos 1 and 2). *Rotarod test*: Motor coordination and balance were assessed by rotarod tests. Male *Ngly1*^−/−^ rats showed significantly reduced latency before falling at 5 weeks of age (Fig. 2B). At 13 weeks of age, male *Ngly1*^−/−^ rats had difficulty staying on the rod at all test speeds (Fig. 2B). Female *Ngly1*^−/−^ rats also started to have difficulty maintaining balance on the rod at 5 weeks of age and showed a progressive decline in rotarod performance (Supplementary Material, Fig. S1B). At 29 weeks of age, female *Ngly1*^−/−^ rats could not stand on the rod in the same way as male *Ngly1*^−/−^ rats (Supplementary Material, Fig. S1B). *Gait analysis*: *Ngly1*^−/−^ rats showed obvious gait abnormalities (Supplementary Material, Video 3). In order to further characterize the motor phenotype of *Ngly1*^−/−^ rats, their gait behavior was analyzed at several ages. *Ngly1*^−/−^ rats showed gait abnormalities, including a wide-based ataxic gait (Fig. 2C, left). *Ngly1*^−/−^ rats had significantly shorter stride lengths and increased stance ratio compared with WT rats at the ages of 8 and 29 weeks (Fig. 2C, right). *Grip-strength test*: The grip-strength of forelimbs or forelimbs and hindlimbs was significantly reduced in both male and female *Ngly1*^−/−^ rats when compared with WT rats (Figs 2D and E, Supplementary Material, Fig. S1C and D). Consistent with the results of grip-strength tests, the weights of the tibialis anterior and gastrocnemius muscles of *Ngly1*^−/−^ rats were also decreased at 15 and 29 weeks of age (Supplementary Material, Fig. S4A and B). Minor muscle atrophy was also observed in some *Ngly1*^−/−^ rats at 29 weeks of age (Supplementary Material, Fig. S4C). *Locomotor activity test*: *Ngly1*^−/−^ rats showed significantly lower activity than WT rats during both the light and dark periods. Collectively, these results suggest that motor function in *Ngly1*^−/−^ rats is impaired from an early age.

### *Ngly1* ^−/−^ rats showed impaired spatial learning

The spatial learning of 14-week-old *Ngly1*^−/−^ and WT rats was evaluated in a Morris water maze. During the hidden platform training, the latency to enter into the hidden platform (goal latency) and total swimming distance were measured.

**Figure 3:**
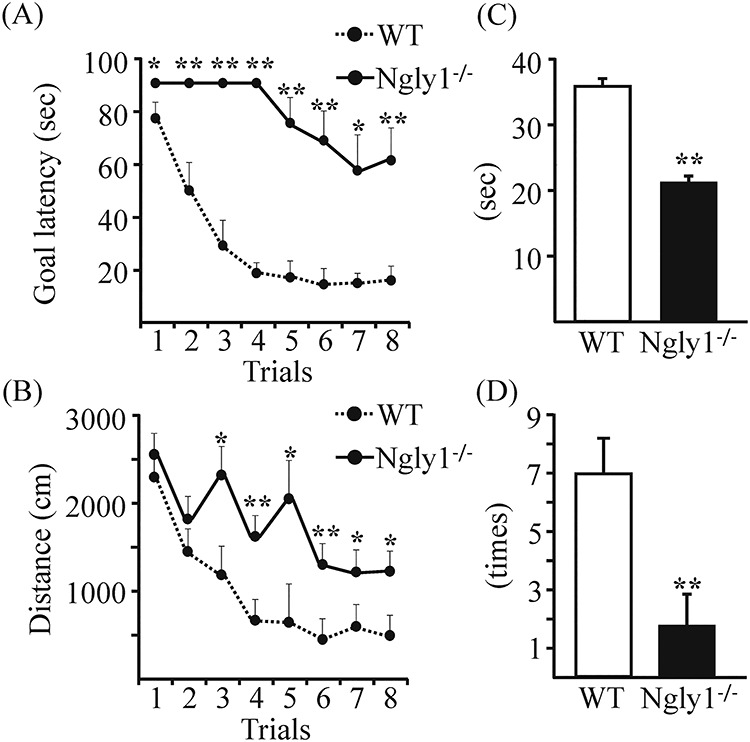
*Ngly1* ^−/−^ rats showed impaired spatial learning in Morris water maze tests. (**A, B**) Performance in eight trials during the acquisition stage of *Ngly1*^−/−^ and WT rats. (A) Goal latency to reach the hidden platform (second). (B) Path length during each trial (centimeters). (**C, D**) Probe tests in the next day of the final acquisition trial. (C) Time spent in the quadrant (seconds). (D) The number of quadrant entries (mean ± SEM). The number of rats examined is 10 each. Asterisks indicate ^*^^*^P < 0.01, ^*^P < 0.05.

There was no significant difference in the swimming speed between WT and Ngly1^−/−^ rats (WT; 29.8 ± 1.51 cm/s, Ngly1^−/−^; 28.1 ± 2.50 cm/s) in the first trial of the hidden platform training, which reflects confounding factors other than the learning abilities. There was a significant difference in the goal latency and total swimming distance between age-matched *Ngly1*^−/−^ and WT rats (Fig. 3A and B), although both *Ngly1*^−/−^ and WT rats learned the hidden platform task and exhibited decreasing goal latency across trials (Fig. 3A). This result suggests that rats acquired the task, but *Ngly1*^−/−^ rats exhibited impaired performance compared with WT rats. During the probe trial, *Ngly1*^−/−^ rats demonstrated significantly impaired performance compared with WT rats, as reflected by a lower time spent in the training quadrant and a decreased number of training quadrant entries (Fig. 3C and D).

### Pathological abnormalities in the central nervous system (CNS) of *Ngly1*^−/−^ rats

**Figure 4:**
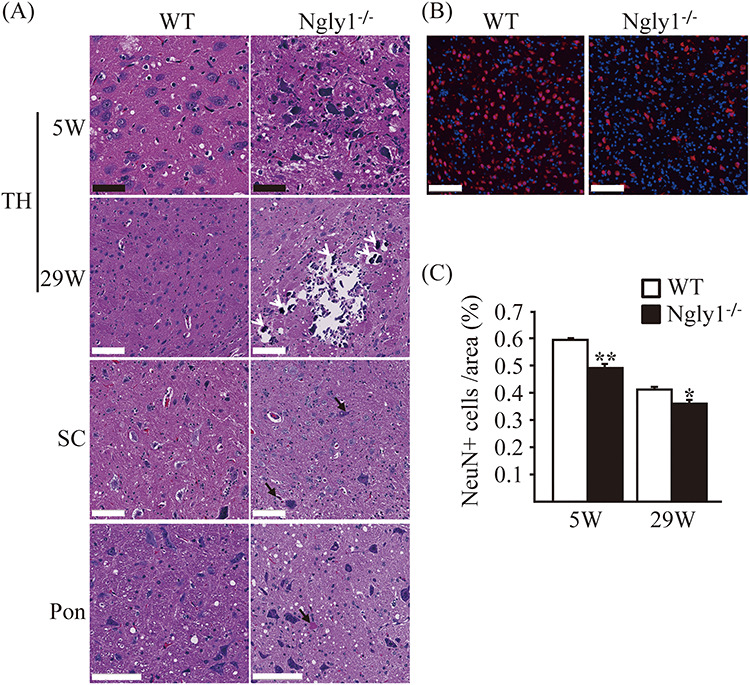
Neuronal degeneration in the central nervous systems of *Ngly1*^−/−^ rats. (**A**) H&E-stained sections of the thalamus (TH), spinal cords (SC) and pons from *Ngly1*^−/−^ and the WT rats at 5 and 29 weeks of age. Black arrows indicate intra- and extracellular eosinophilic inclusion bodies and white arrows indicate mineralization. Black scale bar 50 μm, white scale bar 100 μm. (**B**) Immunohistochemistry of thalamus from *Ngly1*^−/−^ and the WT rats at 5 weeks of age, stained with an anti-NeuN, a mature neuron marker (red). Nuclei were stained with DAPI. Scale bar 100 μm. (**C**) The number of NeuN-positive cells in VPM/VPL, VM and VL regions of the thalamus in *Ngly1*^−/−^ and the WT rats at 5 and 29 weeks of age. Values represent mean ± SEM (*n* = 6–10). Asterisks indicate ^*^P < 0.05 and ^*^^*^P < 0.01.

To elucidate the pathological mechanisms of NGLY1 deficiency, we carried out a comprehensive histological examination of the liver, kidneys, heart, spleen, lungs, eyes, intestines, skin, skeletal muscles (anterior tibialis and gastrocnemius muscle), brain, spinal cord, dorsal root ganglion, sciatic nerves, harderian gland, lacrimal glands, ovaries and uterus in *Ngly1*^−/−^ and WT rats at 5 and 29 weeks of age. We tried to identify pathological abnormalities, which could be associated with neurodegenerative phenotypes in *Ngly1*^−/−^ rats. Histological examination revealed pathological abnormalities in the central and peripheral nervous systems of *Ngly1*^−/−^ rats, but no prominent abnormality was apparent in non-neuronal tissues from our examinations. Histological examination in each region of the brain, including the cerebral cortex, hippocampus, striatum, cerebellum, brainstem, thalamus, hypothalamus, amygdala, pyramids, pons, midbrain and spinal cord, was carried out. Although the *Ngly1* gene is expressed ubiquitously in the entire CNS, the effects of *Ngly1* deficiency on the CNS are selective, and histological abnormalities were most prominent in the lateral and medial parts of the ventral posterior nucleus (VPM/VPL) and the ventral lateral (VL) nucleus of the thalamus in *Ngly1*^−/−^ rats. *Ngly1*^−/−^ rats showed necrotic lesions and mineralization in these regions on hematoxylin and eosin (H&E) section (Fig. 4A). We also found a number of eosinophilic inclusion bodies in the thalamus, spinal cord and pons of *Ngly1*^−/−^ rats on H&E section (Fig. 4A). Immunohistochemical straining with an antibody against mature neurons demonstrated partial loss of neurons in these regions of the thalamus (Fig. 4B and C). Although the accumulation of ubiquitinated proteins was observed in the spinal cord of *Ngly1*^−/−^ rats (Supplementary Material, Fig. S5A), the number of cells positive for choline acetyltransferase, a motor neuron marker in the spinal cord (L2−L4), was not significantly decreased in *Ngly1*^−/−^ rats (Supplementary Material, Fig. S5B). We investigated whether *Ngly1* deficiency induces apoptosis in neurons by immunohistochemistry of cleaved caspase-3. However, cleaved caspase-3-positive neurons were not detected in these regions of *Ngly1*^−/−^ rats (Supplementary Material, Fig. S6). These results suggest that *Ngly1* deficiency in rats induces premature degeneration of neurons in the thalamus through an apoptosis-independent pathway or, alternatively, through apoptosis not detectable by our assays.

### Axonal degeneration in the sciatic nerve of *Ngly1*^−/−^ rats

**Figure 5:**
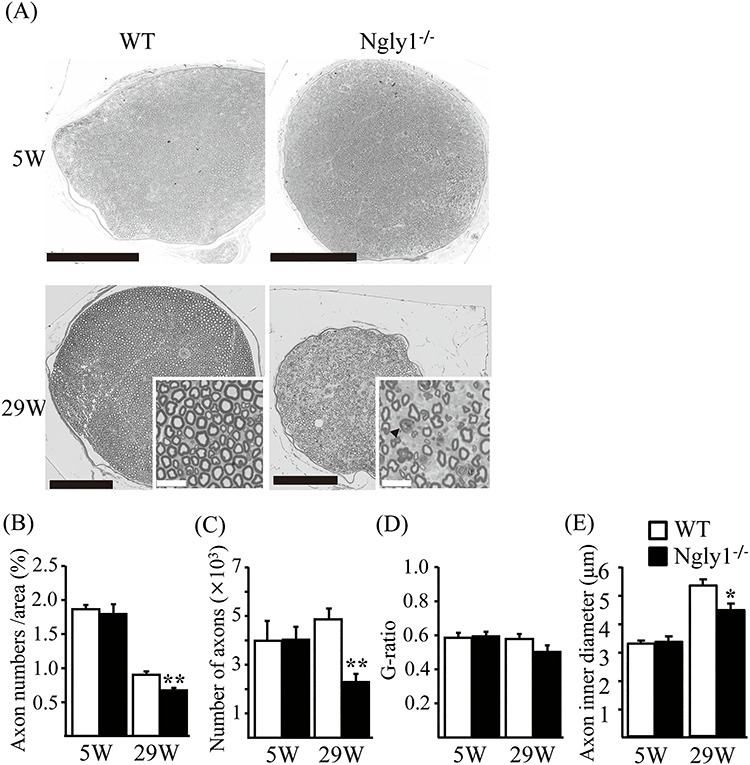
Axonal degeneration of older *Ngly1*^−/−^ rats. (**A**) Toluidine-blue-stained plastic semi-thin sections of sciatic nerve of *Ngly1*^−/−^ rats and WT rats at 5 or 29 weeks of age. Arrows indicate the myelin ovoid. Black scale bar 250 μm, white scale bar 25 μm. (**B–E**) Quantification of total number of axons (B), the density of axons (C), g-ratio (D) and average axon inner diameter (E) per nerve. Values represent mean ± SEM (*n* = 5–10). Asterisk, ^*^^*^P < 0.01, ^*^P < 0.05 (Student’s *t*-test).

In addition to the CNS, the peripheral nerve abnormalities in *Ngly1*^−/−^ rats were examined. We performed a histological analysis of the sciatic nerves in *Ngly1*^−/−^ rats at different ages. Toluidine blue-stained semi-thin sections of the sciatic nerve in *Ngly1*^−/−^ rats appeared normal at 5 weeks of age (Fig. 5A) and showed that there are no differences in the number of axons and the extent of myelination (Fig. 5B and C). At 29 weeks of age, the density of myelinated axons was clearly decreased in *Ngly1*^−/−^ rats (Fig. 5A) compared with WT rats (Fig. 5B and C), as was the number of axons, whereas there was no significant difference in the g-ratio between *Ngly1*^−/−^ and WT rats (Fig. 5D). At 29 weeks old, *Ngly1*^−/−^ rats showed significantly decreased axon inner diameters (indicating loss of large-diameter fibers), a relative increase in small-diameter fibers, and the presence of intra-axonal structure, including a myelin ovoid, which is characteristic of axonal degeneration (Fig. 5A and E). These results suggest that *Ngly1* deficiency in rats would cause axonal loss but does not affect myelination in sciatic nerves.

### Astrogliosis and microgliosis in the thalamus of *Ngly1*^−/−^ rats

**Figure 6:**
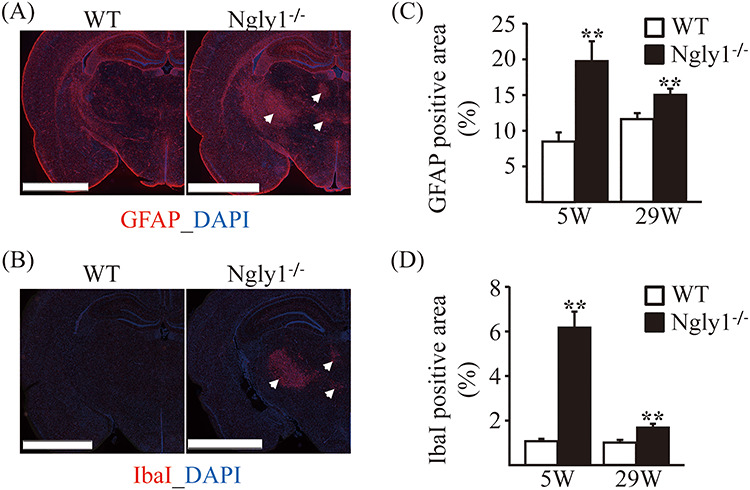
Glial activation in thalamic VPL/VPM, VM and VL regions of *Ngly1*^−/−^ rats. Immunohistochemistry for GFAP in thalamus of WT and *Ngly1*^−/−^ rats at 5 and 29 weeks of age, showing an increase in area of reactive astrocytes. (**A, B**) Immunohistochemistry for GFAP (A) and IbaI (B) in the thalamus of WT and *Ngly1*^−/−^ rats at 5 weeks of age. Scale bar 2.5 mm. Arrows indicate the regions with gliosis in *Ngly1*^−/−^ rats. Nuclei were stained with DAPI. (**C, D**) Quantitative analyses show the area occupied by the GFAP (C) or IbaI (D) positive areas in thalamus of WT and *Ngly1*^−/−^ rats. Values represent mean ± SEM (*n* = 6). Asterisks indicate ^*^^*^P < 0.01.

**Figure 7:**
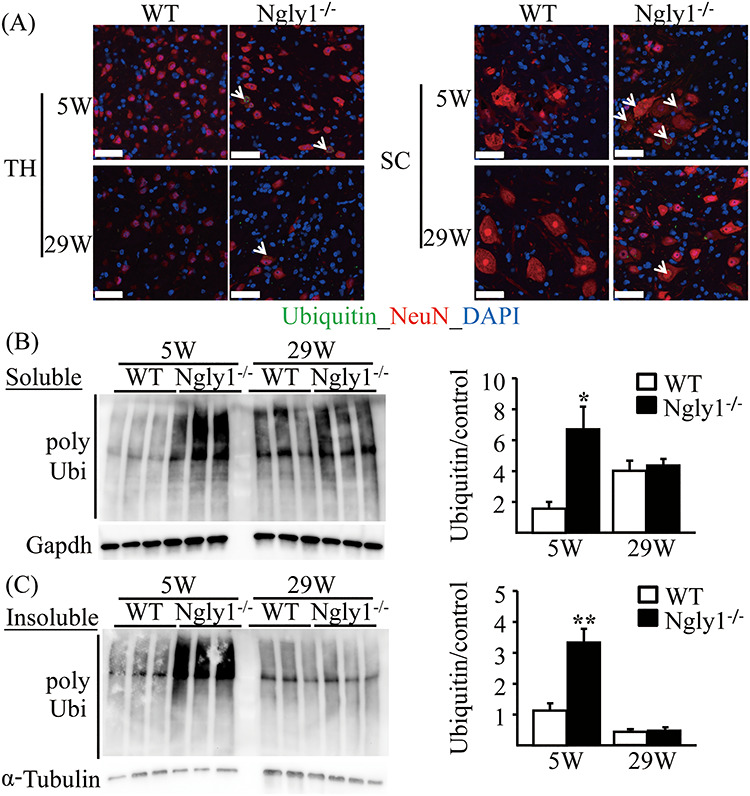
Accumulation of ubiquitinated proteins in the central nervous systems of *Ngly1*^−/−^ rats. (**A**) Ubiquitin-positive inclusions in neurons of *Ngly1*^−/−^ rats. Immunohistochemistry of thalamus (TH) and spinal cord (SC) sections from *Ngly1*^−/−^ and the WT rats at 5 and 29 weeks of age, stained with an anti-ubiquitin antibody and NeuN, a mature neuron marker. Arrows indicate ubiquitin-positive neurons. Nuclei were stained with DAPI. Scale bar 50 μm. (**B, C**) Accumulation of Triton-X-100-soluble (B) and insoluble (C) polyubiquitinated proteins in spinal cords of *Ngly1*^−/−^ and WT rats. The total protein extracts were separated from the spinal cords of the rats and were separated into Triton-X-100-soluble and Triton-X-100-insoluble fractions and analyzed by immunoblotting using anti-polyubiquitinated antibodies (top) and anti-GAPDH or α-tubulin antibodies (bottom; loading control). Semi-quantitative analyses by densitometry were carried out (B, C). Values represent mean ± SEM (*n* = 3). Asterisks indicate ^*^P < 0.05 and ^*^^*^P < 0.01.

Because reactive astrocyte and microglial activation are often regarded as indications of neural toxicity or neuronal death, we quantified the level of the glial fibrillary acidic protein (GFAP) expression using immunofluorescence staining. Compared with WT rats, *Ngly1*^−/−^ rats developed enhanced GFAP immunoreactivity in the VPM/VPL and VL regions of the thalamus (Fig. 6A), in which histological abnormalities on H&E section were observed. Consistent with the astrogliosis, microglial activation was also observed, as indicated by the enhanced immunoreactivity of ionized calcium-binding adaptor molecule 1 (IbaI) in the same regions of the thalamus of *Ngly1*^−/−^ rats (Fig. 6B). Astrogliosis and microgliosis in the thalamus of *Ngly1*^−/−^ rats were more prominent at 5 weeks of age than at 29 weeks of age (Fig. 6C and D). At 2 weeks of age, *Ngly1*^−/−^ rats showed no increased GFAP or IbaI signals in the region when compared with WT rats (Supplementary Material, Fig. S7A). Astrogliosis and microgliosis were also not observed in the spinal cords of *Ngly1*^−/−^ rats at 5 and 29 weeks of age in our examinations (Supplementary Material, Fig. S8), while the accumulation of eosinophilic inclusions and ubiquitinated proteins was observed (Figs 4A and 7A).

### Accumulation of ubiquitinated proteins in the CNS of *Ngly1*^−/−^ rats

NGLY1 is thought to play an important role in the efficient degradation of misfolded glycoproteins during ERAD (1–5). Accumulation of ubiquitinated proteins is characteristic of impaired ERAD systems. We examined protein aggregation in the CNS using an antibody against ubiquitin, a marker of misfolded proteins. Ubiquitin-positive proteins frequently accumulated in the cytoplasm of cells in the thalamus and spinal cord of *Ngly1*^−/−^ rats, but this was not observed in WT rats (Fig. 7A). We observed ubiquitin-positive proteins exclusively in cells positive for the neural cell marker Map2, suggesting that ubiquitin-positive proteins accumulate only in neurons and not in glial cells (Supplementary Material, Fig. S9). Immunohistochemical analysis indicated that the ubiquitin-positive signals are stronger in 5-week-old *Ngly1*^−/−^ rats than in 29-week-old *Ngly1*^−/−^ rats (Fig. 7A). Ubiquitin-positive cells were also detected in the thalamus of *Ngly1*^−/−^ rats at 2 weeks of age (Supplementary Material, Fig. S7B). These data may indicate that accumulation of misfolded proteins in *Ngly1*^−/−^ rats starts at a younger age. We carried out immunoblotting to see if the increased ubiquitin signals came from polyubiquitinated proteins. We also fractionated the brain lysate into TritonX-100-soluble and TritonX-100-insoluble proteins to confirm whether insoluble inclusion body formation would occur after the accumulation of soluble poly-ubiquitinated proteins. At 5 weeks of age, the amount of polyubiquitinated proteins from the spinal cords of *Ngly1*^−/−^ rats was apparently higher than in WT rats in both Triton-soluble and Triton-insoluble fractions (Fig. 7B and C). However, there was no difference in the accumulation of Triton-soluble and Triton-insoluble polyubiquitinated proteins between *Ngly1*^−/−^ and WT rats at 29 weeks of age (Fig. 7B and C).

### ER stress in the brains of *Ngly1*^−/−^ rats

Accumulated ubiquitinated proteins are a hallmark of an impaired ERAD system and ER stress. To confirm whether the loss of NGLY1 causes the ER stress condition in the brain, the protein levels of ER stress markers eIF2, p-eIF2, GRP78 and XBP1 were analyzed by immunoblotting. There was no detectable difference between WT and *Ngly1*^−/−^ rats at postnatal day 1 (P1) or at 2 and 34 weeks of age in the protein levels of ER stress markers (Supplementary Material, Fig. S10). Immunohistochemistry revealed that the expression of XBP1 did not alter in the VPM/VPL regions of the thalamus with ubiquitin-positive proteins in *Ngly1*^−/−^ rats (data not shown).

### NRF1-regulating gene expression in the brain of *Ngly1*^−/−^rats

Previous studies have revealed that NGLY1 deglycosylates and regulates a transcription factor, NRF1, which responds to a number of cellular stresses, including proteasomal stress, and upregulates transcription of proteasomal subunits and general stress response genes (23,24). To verify the relationship between the pathological abnormalities in the CNS of *Ngly1*^−/−^ rats and NRF1-regulating signals, the protein levels of proteasome subunits and deubiquitinating enzymes (DUBs), which are regulated by NRF1, were analyzed by immunoblotting in the brains of WT and *Ngly1*^−/−^ rats at P1 and 2 and 34 weeks of age. There was no detectable difference in the protein levels of NRF1 and NRF1-regulating proteasome subunits (PSMB6 and PSMA5) and DUBs (USP15 and USP9X) between WT and *Ngly1*^−/−^ rats (Supplementary Material, Fig. S11).

## Discussion

The mechanisms underlying neurological symptoms in NGLY1 deficiency are poorly understood, and no effective therapy is available. Animal models that recapitulate the clinical signatures of NGLY1-deficient patients are needed to elucidate the relationship between the *NGLY1* gene and the disease mechanism. The present work revealed that *Ngly1*^−/−^ rats represent the first systemic *Ngly1*-deficient model that develops symptoms that are characteristic of human NGLY1-deficient patients, including developmental delay, motor dysfunction, learning disability and other neurological phenotypes (Supplementary Material, Table S2). Axonal degradation in the sciatic nerves, an indicator of peripheral neuropathy, which is frequently observed in human patients, was also seen in *Ngly1*^−/−^ rats. Additionally, we revealed that *Ngly1*^−/−^ rats had clear histological signs of neurodegeneration, including accumulation of ubiquitinated proteins, necrotic lesions, mineralization, intra- and extracellular eosinophilic bodies, astrogliosis, microgliosis and significant loss of mature neurons, as with other neurodegenerative disease models (32).

Here, we verified the construct and face validity of *Ngly1*^−/−^ rats as a model animal for NGLY1 deficiency. In this study, the exon 11, exon 12 and a 3′ flanking region of the rat *Ngly1* genome were deleted by genome editing technologies. To date, this type of deletion has not been identified in human patients. Most mutations in human patients lead to the reduced mRNA expression of NGLY1 and the absence of detectable NGLY1 protein, although there is an exception (10), suggesting that most disease-causing mutations in human NGLY1 deficiency could cause loss of NGLY1 function. Rat NGLY1 proteins were also not detectable in the brains of *Ngly1*^−/−^ rats, as is the case with most patient fibroblasts. Thus, we believe that the *Ngly1*^−/−^ rats in this study could be of some value for understanding the pathophysiology of NGLY1 deficiency, even if this allele has not been found in patients. *Ngly1*^−/−^ rats recapitulated a variety of symptoms seen in human patients, such as developmental delay, movement disorder, somatosensory impairment, scoliosis and lower brain weight (6–9,12,13). The frequency of onset of the above symptoms varied in *Ngly1*^−/−^ rats. As with human NGLY1 deficiency, all *Ngly1*^−/−^ rats showed a motor deficit, whereas only some *Ngly1*^−/−^ rats had scoliosis. Therefore, *Ngly1*^−/−^ rats represent an experimentally reproducible *in vivo* model of NGLY1 deficiency that can be utilized to evaluate therapeutic options and to study the cellular and molecular disease mechanisms in this disease.

Identifying the regions with pathological abnormalities in *Ngly1*^−/−^ rats is essential for precisely understanding the pathological mechanisms of NGLY1 deficiency and developing therapeutic options for NGLY1 deficiency. In this study, we carried out a comprehensive histological study of 18 tissues. Histological examination identified pathological abnormalities in the central and peripheral nervous systems of *Ngly1*^−/−^ rats. We examined each region of the brain, including the cerebral cortex, hippocampus, striatum, cerebellum, brainstem, thalamus, hypothalamus, amygdala, pyramids, pons and midbrain. Striking pathological features of *Ngly1*^−/−^ rats included necrotic lesions, intra- and extracellular eosinophilic bodies, astrogliosis, pronounced microglial activation, mineralization and significant loss of neurons within the VPM/VPL, VL and ventromedial (VM) nuclei of the thalamus. The regional specificity of those changes was evident. They did not occur in other tissues, other regions of the brain or the neighboring thalamic regions in our examinations. These nuclei in the thalamus send efferent projections to regions, including the motor cortex, helping with coordination, planning and learning of movement (33). The VL and VM nuclei are classified as motor regions of the thalamus and function as a hub to transmit signals from the basal ganglia and cerebellum to the motor cortex (33). Previous studies have revealed that motor thalamic lesions impair acquisition in the rotarod test and the Morris water maze, indicating that thalamic motor nuclei participate in the acquisition of sensorimotor and spatial learning (33). These observations are consistent with our results from rotarod tests and Morris water maze tests, suggesting that pathological abnormalities in these thalamic regions could contribute to motor dysfunction and impaired spatial learning in *Ngly1*^−/−^ rats. The VPM/VPL nuclei of the thalamus are also central to the relay of sensory information from the hindbrain and spinal cord to the sensorimotor cortex. The presence of pathological abnormalities in these regions could affect somatosensory impairment and result in reduced auditory and pain reflex in *Ngly1*^−/−^ rats. To confirm whether the thalamus could be an important pathological target in Ngly1^−/−^ rats, we need to observe the thalamus of NGLY1-defcient patients. However, we do not have any data in patients that specifically implicates the thalamus defect. Further studies by MRI in human patients will be needed.

To elucidate the molecular mechanisms of NGLY1 deficiency, we focused on the ERAD and ubiquitin-proteasome systems. The ubiquitin-proteasome system has been implicated in several neurodegenerative diseases based on the presence of deposits consisting of ubiquitin-positive proteins in affected neurons (32). NGLY1 is thought to play a pivotal role in ERAD processes, deglycosylating misfolded glycoproteins before they are degraded by the proteasome (1–5). Ubiquitin-positive proteins were more frequently observed in the neurons of the thalamus and spinal cords of *Ngly1*^−/−^ rats. Neurons are generally sensitive to ER stress because of the unique characteristics of fully differentiated neurons, which are highly metabolically demanding. However, *Ngly1*^−/−^ rats showed no difference in the protein levels of ER stress markers at a variety of ages when compared with WT rats (Supplementary Material, Fig. S10). In addition to deglycosylation of misfolded proteins in the ERAD process, NGLY1 appears to be important for regulating cellular signaling in ERAD by deglycosylating certain molecules, erythroid 2 like 1 (NFR2L1, also known as NRF1) (19,23,24). If NGLY1 function is absent, NRF1 remains glycosylated and cannot properly regulate its target stress response genes (23,24). *Ngly1*^−/−^ rats showed no difference in the protein levels of proteasome subunits and DUBs that are regulated by NRF1 when compared with WT rats (Supplementary Material, Fig. S11). These observations are consistent with the previous observation of hepatocyte-specific knockout mice (25), where no transcriptional signature indicating the defective NRF1 activity was evident. These results suggest that ER stress and the NRF1 axis should not be a major cause for the pathogenesis of these mutant rats after weaning. It should be noted that our rats are, in sharp contrast to the mouse model, outbred, and they still showed a reduced birth rate as well as premature death before weaning (Fig. 1E). It is, therefore, tempting to speculate that this NRF1 defect would have already had a great effect on phenotypes at an embryonic stage and that rats that survived after weaning may be intrinsically resistant to the NRF1 defect. Further studies of animal models at the developmental stage will be required to elucidate the precise molecular mechanisms of disease onset and progression.

Despite being ubiquitously expressed, the regional pathological abnormalities of *Ngly1*-deficient rats reflect a difference in the ability to dispose of misfolded proteins. This selective neuronal vulnerability in *Ngly1*^−/−^ rats remains unclear, although it may involve neuron-specific combinations of dysfunctions in cellular stress and proteostasis pathways (34). Previous studies reported that the VPM/VPL is a particularly vulnerable region with glial activation in neurodegenerative animal models of diverse lysosomal storage disorders (35–40). The VPM/VPL nuclei were used as a paradigm of neuronal rescue in adeno-associated virus-mediated gene transfer treatment for these diseases (41–43). When animal models of Tay-Sachs-related diseases and Sandhoff disease were treated by AAV-mediated therapeutic genes delivery, their neuronal density in the VPM/VPL is also correlated directly with an increased lifespan of them, accompanying the prevention of microgliosis (42,43). Taken together, it is suggested that the number of neurons and activated microglia in the VPM/VPL is a useful indicator or a surrogate marker for neuronal rescue in evaluating therapeutic options in preclinical experiments. However, it is not clear whether the loss of mature neurons and enhanced gliosis in the VPM/VPL lesion could directly contribute to known symptoms of NGLY1 deficiency. Astrogliosis and microgliosis in the thalamus of *Ngly1*^−/−^ rats were observed at 5 and 29 weeks of age, but not at 2 weeks of age (Supplementary Material, Fig. S7).

In summary, the *Ngly1*^−/−^ rat is a valuable systemic *Ngly1* knockout rodent model recapitulating the neurological, histological and behavioral features of human NGLY1 deficiency. Our studies suggest that the thalamus could be an important pathological target in *Ngly1*^−/−^ rats, and this has important implications for understanding the pathogenesis of this disease.

## Materials and Methods

### Animals

All animal care procedures and experiments conformed to the Association for Assessment and Accreditation of Laboratory Animal Care guidelines and were approved by the Experimental Animal Care and Use Committee of Takeda Pharmaceutical Company Limited. All rats were housed in individual cages in a room with controlled temperature (23°C), humidity (55%) and lighting (lights on from 7:00 a.m. to 7:00 p.m.) and were fed with a normal chow diet (CE2 diet, CLEA Japan) with free access to water. Rats were sacrificed by exsanguination, and a spine curvature in each rat was confirmed when they were sacrificed. Organs were eviscerated and some of them were measured for weights. The spinal cord was dissected, and the thoracic and lumbar segments were identified using the ribs and vertebrae as a guide.

### Generation of *Ngly1*^−/−^ rats

As with *Ngly1* knockout mice (25,26), we deleted the exon 11, exon 12 and a 3′ flanking region of the rat *Ngly1* genome using CRISPR/Cas9 genome editing of a Crl:CD(SD) SD rat, an outbred strain (Fig. 1A), although this type of deletion was not reported in human patients (6–9). The SD rats were obtained from Charles River Laboratories Japan, Inc. Two single-guide RNA (sgRNA) sequences targeting sites upstream (5′-sgRNA; 5′-cagaggaattgtgatagtacagg-3′) and downstream (3′-sgRNA; 5′-ccagttattcataccatggtaaa-3′) of the exon 11, exon 12 and a 3′ flanking region of the rat *Ngly1* genome, respectively, were transcribed *in vitro* using MEGAshortscript Kit (MEGAshortscript T7 Transcription Kit, Life Technologies). A mixture of Cas9 (New England Biolabs, 100 ng/μl), 5′-sgRNA (50 ng/μl) and 3′-sgRNA (50 ng/μl) was microinjected into the fertilized SD rat eggs, and the embryos were transferred to pseudo-pregnant females at the two-cell stage. A genotyping of the *Ngly1*^−/−^ rat was performed by PCR with forward and reverse primers. A forward primer (5′-CAGAGCTTATGTCCACAGAGTCCTT-3′) anneals to the sequence upstream of the target region, and a reverse primer (5′-ACACTATCGTCCTACTCACCTAGCT-3′) anneals to the sequence downstream. These procedures to produce *Ngly1* knockout rats were carried out by Axcelead Drug Discovery Partners, Inc. Heterozygous *Ngly1* knockout rats at F0 generation were backcrossed to SD rats twice. Then, WT and homozygous knockout rats were generated from heterozygote mating. *Ngly1*^−/−^ rats will be deposited in the National BioResource Project—Rat in Japan and are available from the Project.

### The functional observation battery

The FOB was administered in 10-week-old *Ngly1*^−/−^ and WT rats (44). There were four consecutive tests: (1) in the home cage, (2) in the observer’s hand, (3) in the open field and (4) manipulation tests. (1) The rat was observed in its home cage. Observations on activity, stereotypy and abnormal movements (e.g. tremors, convulsions) were recorded. (2) The rat was removed from the home cage and placed in the observer’s hand. Difficulty in removal, ease of handling, muscle tone, fur abnormalities (piloerection), salivation, lacrimation, mucous membranes, pupil size and respiration were monitored. (3) The rat was placed in the open field condition for a 2 min observation period. During this time, the observer noted gait abnormalities, arousal/activity, rearing, defecation, urination, grooming and stereotypy. (4) Manipulation tests were conducted in the open field. The degree of auditory reflex, pain reflex, response to touch, pupillary reflex and approaching behavior were scored.

### Motor function tests

Hindlimb clasping tests: rats were individually lifted by grasping their tails near the base, and their hindlimb positions were observed for 10 s. If both hindlimbs were partially or entirely retracted toward the abdomen for more than 5 s, we defined them as having an abnormal hindlimb clasping reflex.

Rotarod test: motor performance and coordination behaviors were tested using an accelerating rotarod (Muromachi Kikai Co., Ltd, MK610) at different ages. Each rat underwent 2 days of training sessions and a 1-day test session. The first 2 days were used to train the rats in four separate trials of 1 min each, walking at 4 rpm with a 20-min interval between trials. On day three, a test session comprising four separate trials, with a 20 min interval between trials, was run. During the test session, the speed of the rotarod was accelerated from 4 to 40 rpm over the course of 4 min, and the time taken for the rats to fall from the rod was measured. If the rats stayed on the rod until the end of the 4 min trial, a time of 240 s was recorded.

Grip-strength test: during the grip-strength test, an animal was lifted by the tail, and its forelimbs or all limbs were allowed to grasp a metal mesh fixed to a force-electricity transducer (Brain Science Idea Co., Ltd, BS-TM-RM). The animal was gently pulled upward while it grasped the mesh with its forelimbs or all limbs. The maximal force reached immediately before it released the mesh was taken as the grip-strength.

Gait analysis: to assess changes in the gait of rats, gait analysis was carried out. The forepaws and hind paws were coated with two non-toxic water-soluble color inks (forepaws green, hind paws red). The rats were then allowed to walk along a 100-cm-long, 15-cm-wide runway. The floor of the runway was covered with sheets of white paper. The footprints were manually analyzed, and the stride lengths of each paw and the stance ratio (width ratio; hind to forelimbs) were quantified.

Locomotor activity: locomotor activity for an individual rat was detected with an infrared sensor (Neuroscience Inc., NS-AS01) placed above the floor of the home cage (31 × 36 × 17.5 cm). The activity of each 1 min bin was measured by accumulated counts if the animal was active in any area of the cage. The sum of total activity counts for 120 h was calculated for each light and dark period.

### Morris water maze

The water pool was made from gray vinyl chloride and measured 148 cm in diameter, with a water level (at 22°C) reaching 32 cm and with walls 44 cm in height. The escape platform (diameter 12 cm) was made from clear acrylic resin. The maze was contained in a room with various visual cues outside the maze. On days 1–4, acquisition of spatial learning in the Morris water maze was assessed. The rats were placed near the edge and facing the wall successively in five positions (A–E) in a semi-random order during two trials per day with a cut-off period of 90 s. The escape platform was hidden 2 cm beneath the water level in the middle of a quadrant. When reaching the platform, the rats stayed on it for 30 s. Whenever a rat failed to find the platform within the maximally allowed time of 90 s, it was manually placed on it for 30 s. The experimenter was hidden from view but was able to follow the routes taken by the rats by means of a video monitor. The pool was separated into four quadrants displayed on the video monitor, and the escape latencies were measured by video tracking software (EthoVision XT, Noldus Information Technology Inc.). A probe test was conducted on the next day of the final acquisition trial. The rats were placed near the edge of the pool facing the wall in one position. As with the acquisition trials, the number of quadrant entries and the swimming time were measured by the software.

### Histological analysis

For light microscopy analysis, tissues from *Ngly1*^−/−^ rats and WT rats at 5 and 29 weeks of age were dissected and fixed in 10% neutral-buffered formalin. Brains were trimmed coronally at 4 levels (Level 2, 3, 5 and 6) based on the STP position paper [45]. They were paraffin-embedded and sectioned at 4−6 μm thickness and mounted onto slides. Sections were stained with H&E. Sciatic nerves from *Ngly1*^−/−^ rats and WT rats at 5 and 29 weeks of age were dissected and placed in 2% glutaraldehyde/2% paraformaldehyde in 0.1 M PBS (pH = 7.4) at 4°C overnight. After washing them with PBS overnight at 4°C, samples were dehydrated and embedded in the epoxy. For light microscopy, semi-thin plastic embedded sections were prepared and stained with toluidine blue. Each image was analyzed using HALO with the axon module (Indica Labs), which counts and measures axons and myelin areas in nerve cross-sections.

### Immunoblotting analysis

Brains were isolated, desheathed in saline and immediately frozen on dry ice. Whole brain lysates were prepared by homogenizing the tissue in T-PER buffer (Thermo Fisher) with a protease inhibitor cocktail. The protein amount in the lysates was quantified using the BCA Protein Assay Kit (Thermo Fisher). Tissue lysates (10 μg) were collected and resolved in SDS-PAGE (Bio-Rad) and transferred onto polyvinylidene fluoride membranes (Bio-Rad) according to the manufacturer’s instructions. Membranes were incubated with a primary antibody followed by incubation with horseradish peroxidase (HRP)-conjugated secondary antibody. And then these were visualized with the ChemiDoc Imaging System (Bio-Rad) using a western chemiluminescent HRP substrate (Merck Millipore). Preparation of detergent-soluble and detergent-insoluble fractions in rat spinal cords was also described previously (46).

### Immunostaining analysis

For immunohistochemical analysis, all tissue sections were subjected to antigen retrieval using the microwave method (in Tris-EDTA buffer (pH = 9) for 15–20 min). After blocking, sections were incubated with primary antibodies overnight at 4°C, followed by 1 h incubation with fluorescently labeled secondary antibodies.

### Antibodies

The following antibodies were used at the indicated dilutions:

For immunoblotting:

- Rabbit anti-NGLY1 (1:100; ATLAS ANTIBODIES (HPA036825))
- Rabbit anti-Grp78 (1:2000; Novus Biologicals (NBP1-06274SS))
- Rabbit anti-eIF2 (1:1000; CST (#5324))
- Rabbit anti-p-eIF2 (1:1000; CST (#3398))
- Rabbit anti-XBP (1:500; Abcam (ab37152))
- Rabbit anti-PsmB6 (1:1000; CST (#13267S))
- Rabbit anti-PsmA5 (1:2000; CST (#2457S))
- Rabbit anti-Usp9x (1:1000; Novus Biologicals (NBP1–48321))
- Rabbit anti-Usp15 (1:2000; CST (#66310S))
- Rabbit anti-LC3 (1:2000; MBL (PM036))
- Guinea pig anti-p62 (1:1000; PROGEN (GP62-C))
- Rabbit anti-NRF1 (1:1000; CST (#8052))
- Mouse anti-GAPDH (6C5; 1:5000; Millipore (MAB374))
- Mouse anti-α-Tubulin (DM1A; 1:10000; Abcam (ab7291))
- Mouse anti-multi ubiquitin (FK2; 1:100; MBL (D058–3))
- Horse anti-mouse IgG (H+L) antibody, HRP conjugate (1:20000; CST(#7076))
- Donkey anti-rabbit IgG (H+L) antibody, HRP conjugate (1:20000; GE Healthcare (NA934))
- Rabbit anti-Guinea Pig IgG (H+L) antibody, HRP conjugate (1:20000; ThermoFisher(614620))

For immunostaining:

- Rabbit anti-choline acetyltransferase (ChAT) (1:2000; Abcam (ab178850))
- Rabbit anti-GFAP (1:2000; Abcam (ab7260))
- Goat anti-IbaI (1:1000; Abcam (ab5076))
- Rabbit anti-GSTP1 (1:300; GenTex (GTX112695))
- Rabbit anti-MAP2 (1:4000; Abcam (ab183830))
- Rabbit anti-cleaved caspase 3 (1:200; CST (#9661))
- Rabbit anti-NeuN (1:3000; Abcam (ab177487))
- Mouse anti-NeuN (1:1000; BioLegend (SIG-39860))
- Mouse anti-ubiquitin (1B3; 1:200; MBL (MK-11-3))
- Donkey anti-Rabbit IgG (H+L) Highly Cross-Adsorbed antibody, Alexa Fluor 568 conjugate (1:2000; ThermoFisher (A10042))
- Donkey anti-Mouse IgG (H+L) Highly Cross-Adsorbed antibody, Alexa Fluor 488 conjugate (1:2000; ThermoFisher (A21202))
- Donkey anti-Goat IgG (H+L) Cross-Adsorbed antibody, Alexa Fluor 568 conjuate (1:2000; ThermoFisher (A11057))

### Statistical analysis

Data are presented as mean ± SEM. Statistical significance was determined using a Student’s *t*-test to compare two groups at ^*^P < 0.05 and ^*^^*^P < 0.01.

## Supplementary Material

Supplemental_materials_ddaa059

mvi_0063_ddaa059

mvi_0158_ddaa059

mvi_0198_ddaa059
