# Supplementary material for: Ngly1 −/− rats develop neurodegenerative phenotypes and pathological abnormalities in their peripheral and central nervous systems
Source: Hum Mol Genet. 2020 Apr 7;29(10):1635–47. doi: 10.1093/hmg/ddaa059 (PMC7322575; doi:10.1093/hmg/ddaa059)
Supplement: Supplemental_materials_ddaa059 [file supplemental_materials_ddaa059.pdf]

Supplementary Material

Ngly1−/− rats develop neurodegenerative phenotypes and pathological abnormalities in their peripheral and central nervous systems

Makoto Asahina, Reiko Fujinawa, Sayuri Nakamura, Kotaro Yokoyama, Ryuichi Tozawa, and Tadashi Suzuki

Supplemental Table 1, 2
Supplemental figures 1-11
Supplemental video 1-3

Supplemental Table 1 Phenotypes in model organisms without NGLY1.

Species	Strain	Phenotype observed	ref
S. cerevisiae		No obvious defects	1
N. crassa		Temperature-sensitive growth with storong polarity defects	15, 17
D. melanogaster		Severe developmental delay, sensitivity towards proteasome inhibitor	14, 20, 21, 22
C. elegans		Abnormal axon branching, egg-laying behaviour defect, sensitivity towards proteasome inhibitor	16, 19, 24
M. musculus	Ngly1-/- in C57BL/6	Embryonic lethality	26
	Ngly1-/- in C57BL/6 and ICR mixed	Premature death, weight decrease, hindlimb clasping, scoliosis	26
	Engase-/- Ngly1-/- in C57BL/6	Weight decrease, hindlimb clasping, scoliosis	26
H. sapiens		Global develpmental delay, movement disorder, hypotonia	6–9, 12, 13

Supplemental table 2 The comparison talbe between the Ngly1-/- rat model and Ngly1 deficient mouse models.

	Mouse (Ref. 26)			Rat
Genotype	Ngly1-/-	Engase-/- Ngly1-/-	Ngly1-/-	Ngly1-/-
Background	C57BL/6 (B6)	C57BL (B6)	B6_ICR mixed	SD
Phenotypes				
Embryonic lethality	+	-	-	-
Premature death	n/a	-	+	+
Hindlimb clasping	n/a	+	+	+
Weight loss	n/a	+	+	+
Scoliosis	n/a	+	+	+
Motor dysfunction	n/a	n/a	n/a	+
Gait abnormality	n/a	n/a	n/a	+
Spatial learning disability	n/a	n/a	n/a	+
Pathological abnormalities	n/a	n/a	n/a	+

n/a: not available

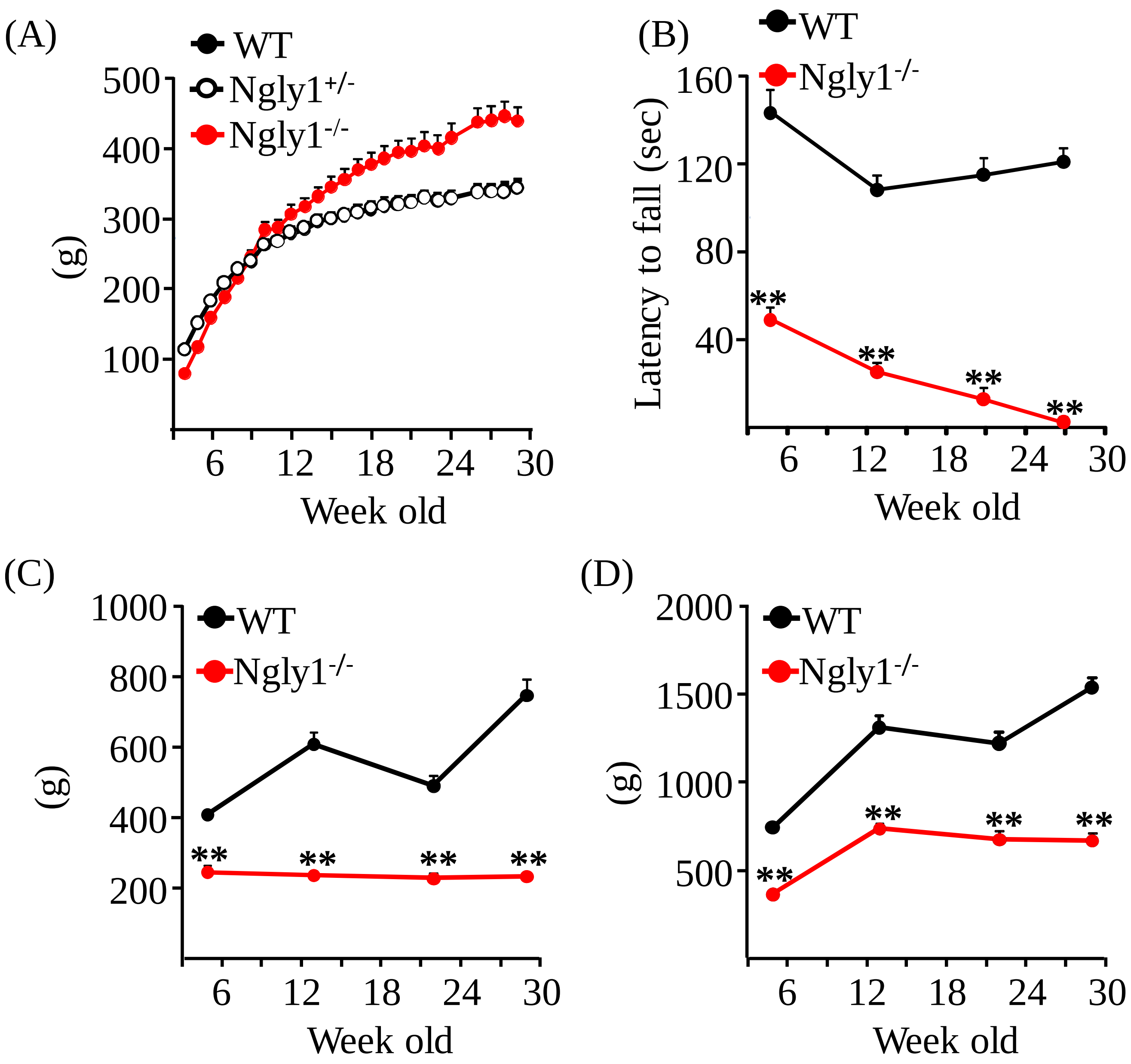

Supplementary Figure 1

Phenotypes of female *Ngly1*−/− rats.

(A) Body weight of *Ngly1*−/−, *Ngly1*−/+, and WT rats. Rats were weighed weekly after weaning. Female *Ngly1*−/− rats showed significantly reduced body weights compared with WT rats until they were 7 weeks of age. They gradually gained weight and showed increased body weights after 13 weeks of age. (B) Rotarod test for motor coordination of *Ngly1*−/− and WT rats. The rotarod test was carried out as with male rats. Female *Ngly1*−/− rats showed a progressive decline in rotarod performance. (C, D) Grip-strength test for forelimb (C) or for forelimb and hindlimb (D) muscle force. Grip strengths of forelimb or forelimb and hindlimb were significantly decreased in female *Ngly1*−/− rats compared with WT rats. Values represent means ± SEM. The number of rats examined was 16 each. Asterisks indicate **P < 0.01, *P < 0.05 (Student's t-test).

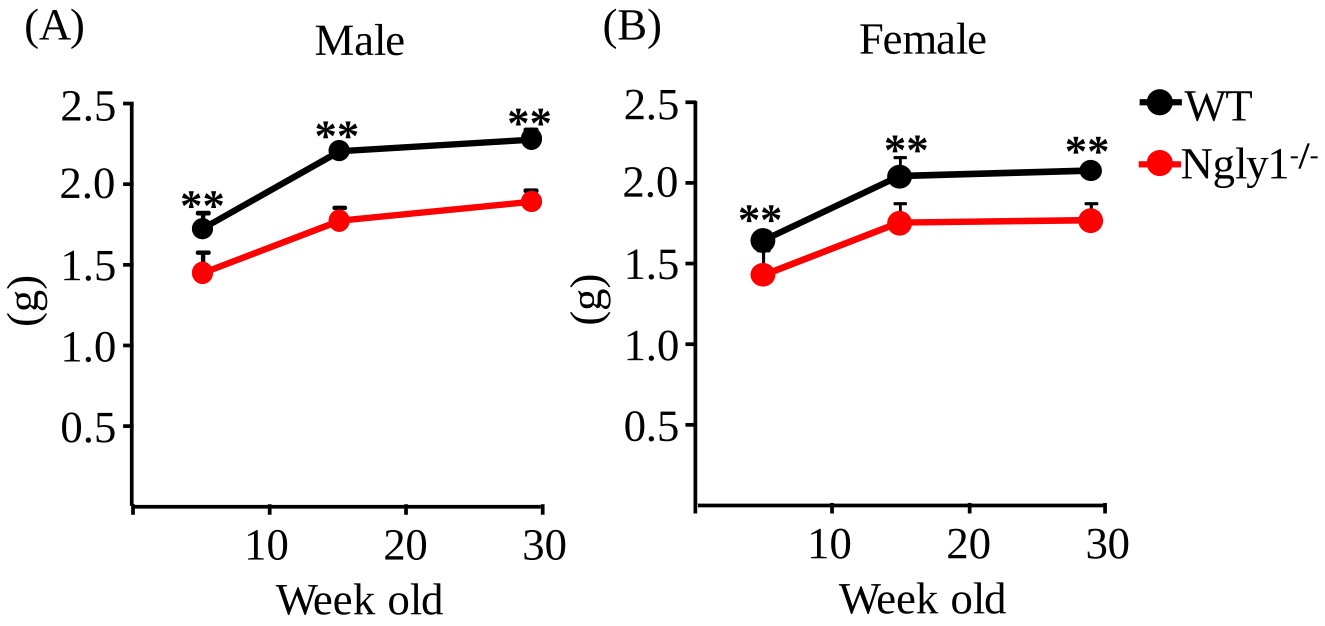

Supplementary Figure 2

Brain weights at 5, 15, 29 weeks of age in male (A) and female (B) rats. *Ngly1*−/− brain weights were significantly lower than those of WT rats. Values represent means ± SEM (n = 6-10, each sex). Asterisks indicate **P < 0.01, *P < 0.05 (Student's t-test).

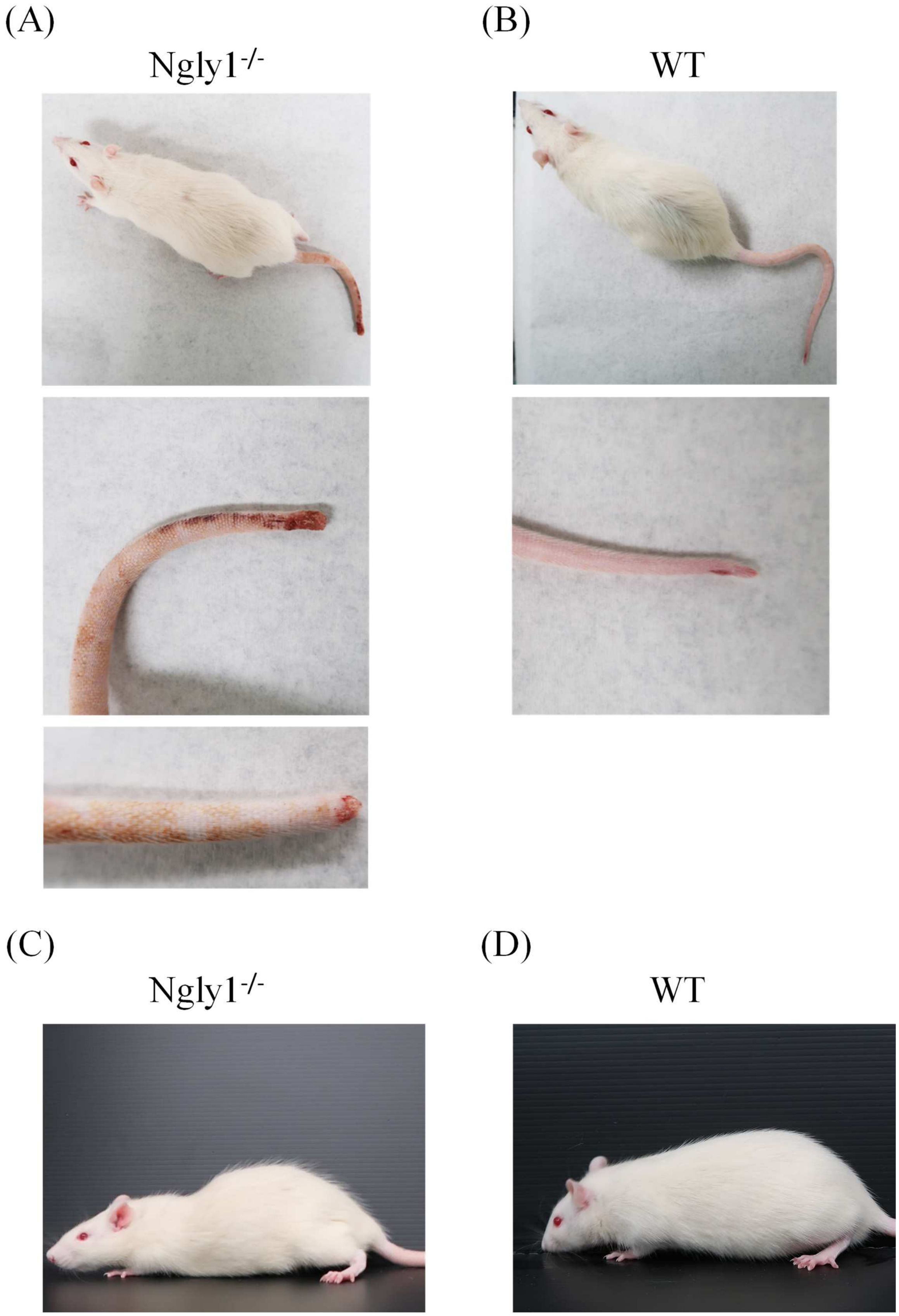

Supplementary Figure 3

Representative tail necrosis in male 15-week-old *Ngly1*−/− rats (A) and age-matched WT rats (B). Representative scoliosis in male 29-week-old *Ngly1*−/− rats (C) and age-matched WT rats (D)

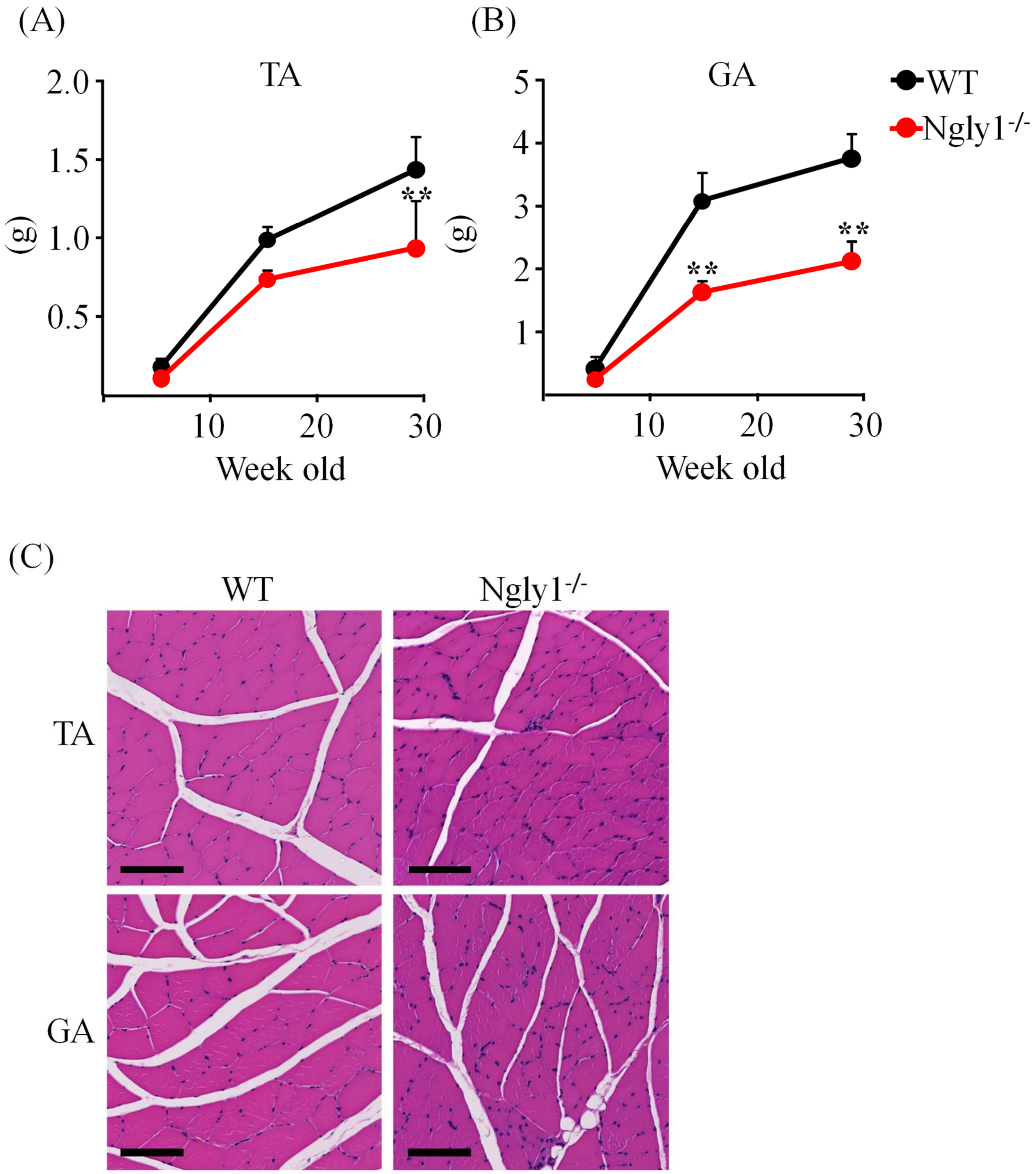

Supplementary Figure 4

(A, B) Weights of tibialis anterior (TA) and gastrocnemius (GA) muscles of *Ngly1*−/− and WT rats at 5, 15, and 29 weeks of age. Values represent means ± SEM (n = 6-10, each sex). Asterisks indicate **P < 0.01 (Student's t-test). (C) H&E-stained sections of TA and GA muscles from *Ngly1*−/− and the WT rats at 29 weeks of age. Scale bar 100 μm.

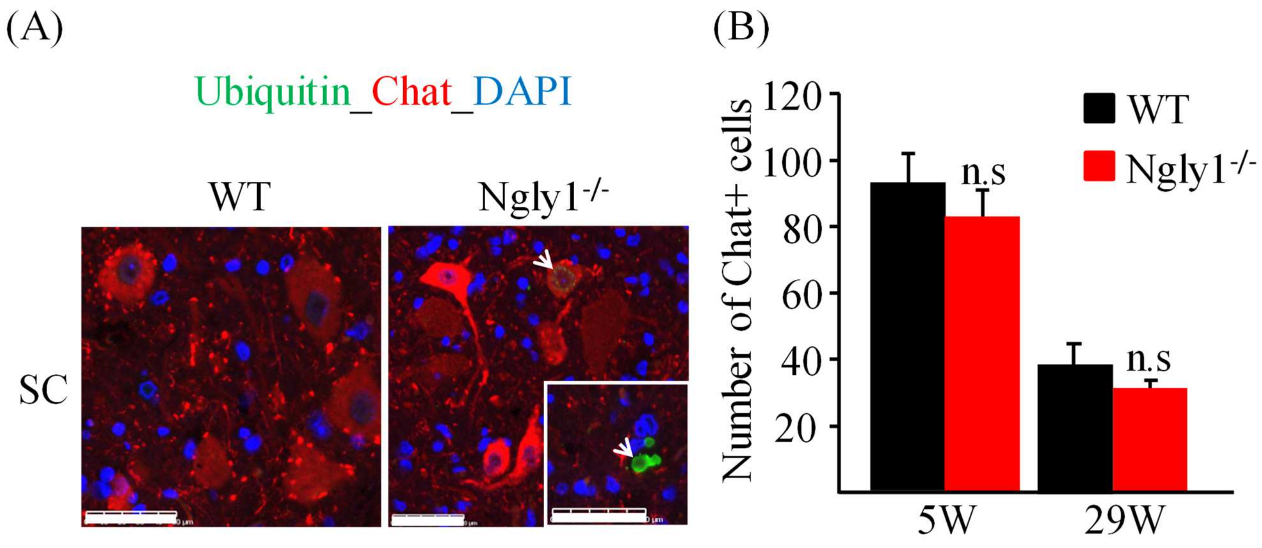

Supplementary Figure 5

(A) Immunohistochemical staining with ubiquitin (green) and motor neurons (red) in spinal cords of 29-week-old rats, using antibodies to ubiquitin and ChAT. The nuclei have been stained with DAPI (blue). Arrows indicate the ubiquitin staining. Scale bar 50 µm. (B) The number of ChAT-positive cells in L2–L4 regions of spinal cords in *Ngly1*−/− and WT rats at 5 and 29 weeks of age. Values represent mean ± SEM (n = 6–10). Asterisks indicate *P < 0.05 and **P < 0.01.

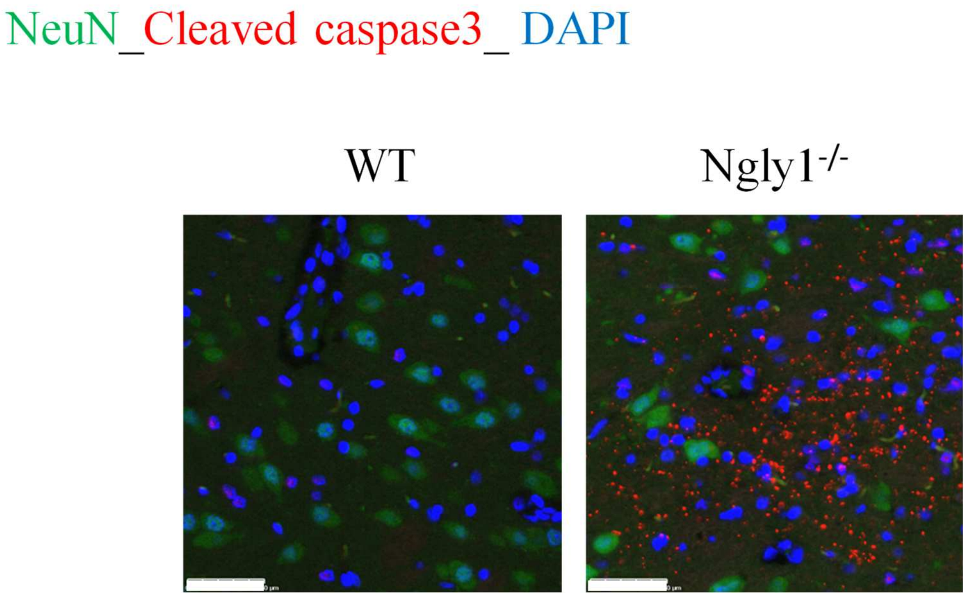

Supplementary Figure 6

Immunohistochemical staining with NeuN (green) and cleaved caspase-3 (red) in the thalamic VPM/VPL regions of 29-week-old rats. The nuclei have been stained with DAPI (blue). Scale bar 50 µm.

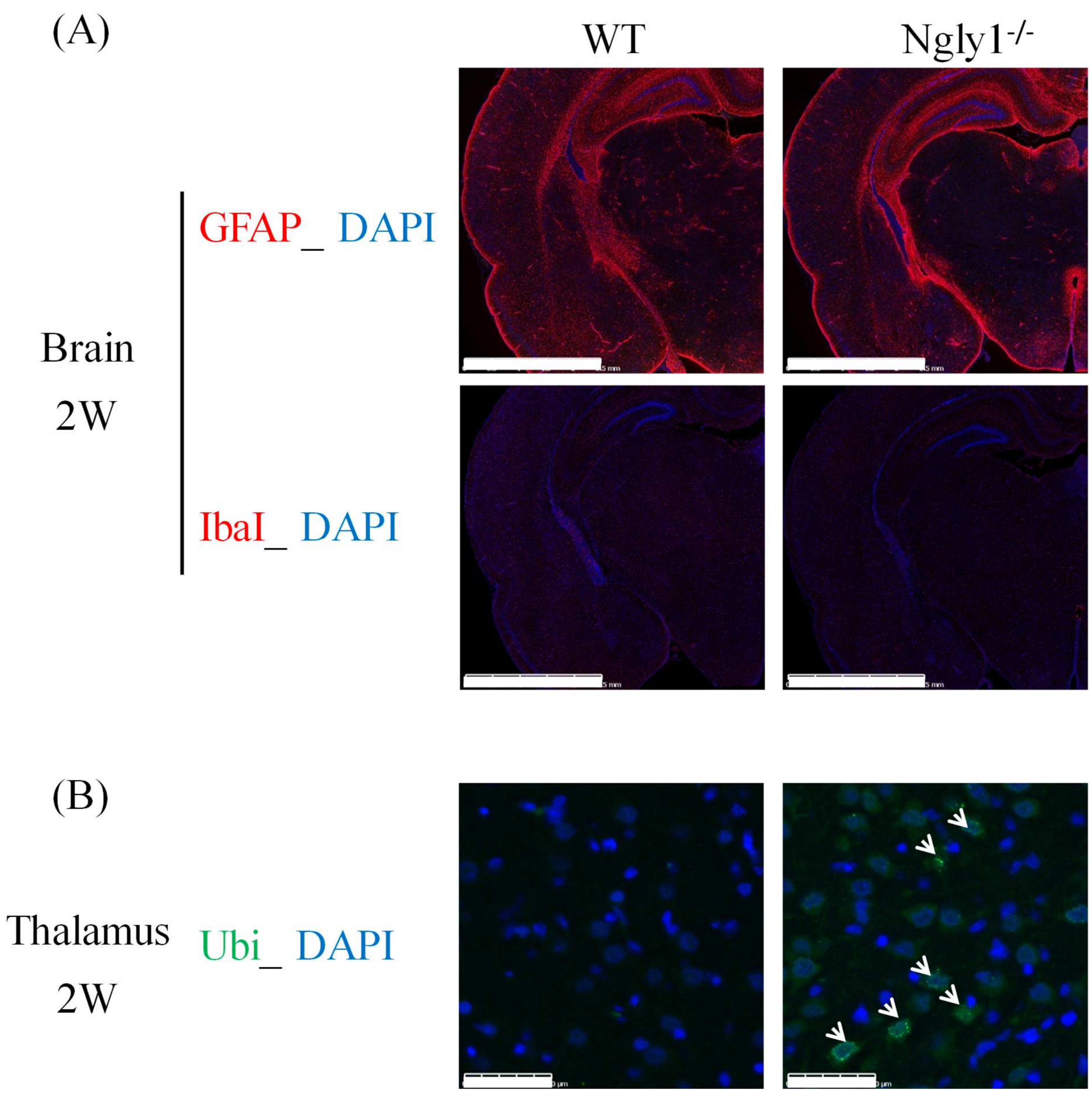

Supplementary Figure 7

(A) Immunohistochemical staining with GFAP or IbaI (red) in the brains of 2-week-old rats. Scale bar 2.5 mm. (B) Immunohistochemical staining with ubiquitin (green) in the thalamus of 2-week-old rats. Scale bar 50 μm. Arrows indicate ubiquitin-positive cells. The nuclei have been stained with DAPI (blue).

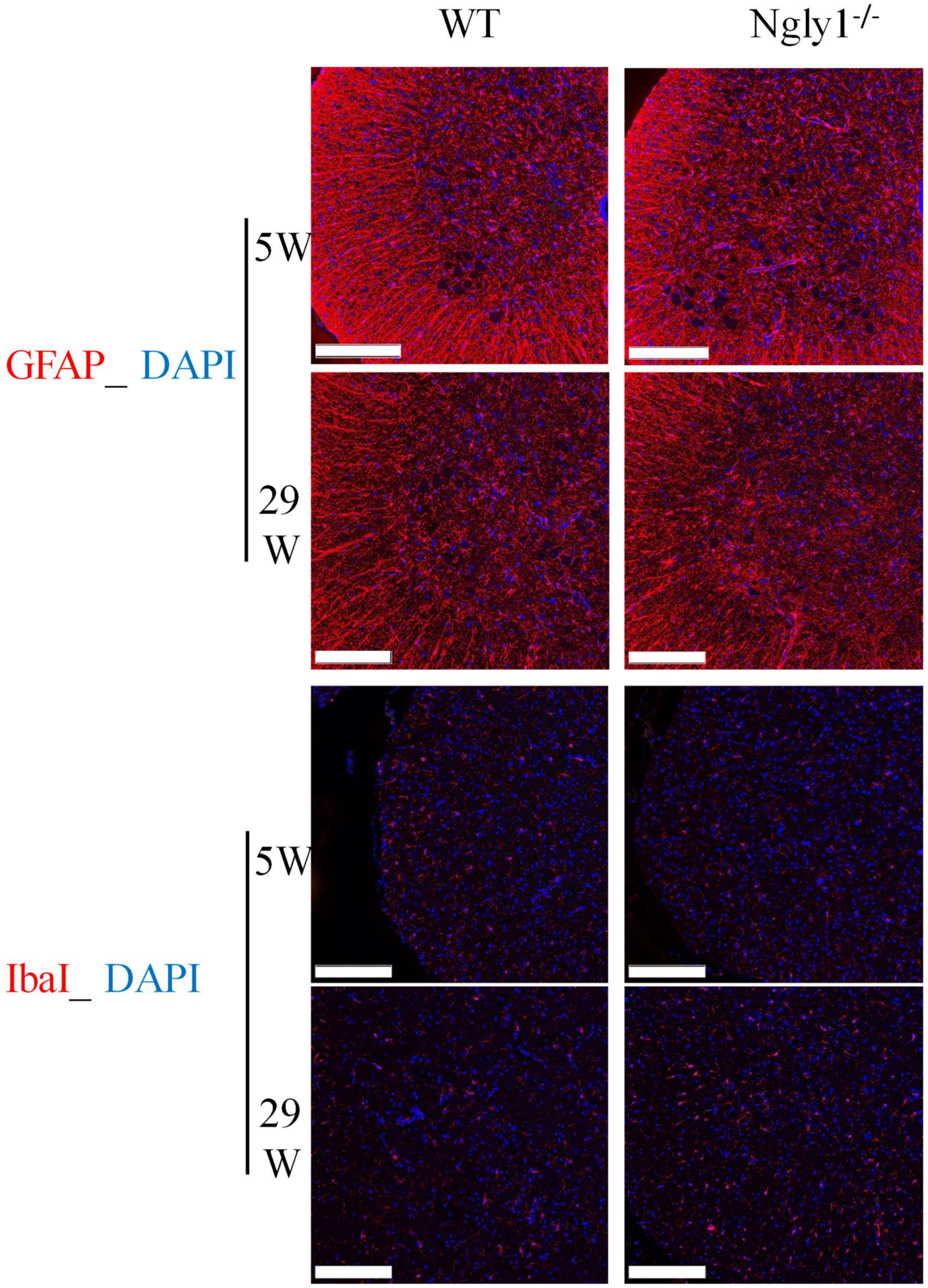

Supplementary Figure 8

Immunohistochemical staining with GFAP or IbaI (red) in spinal cords of 5- or 29-week-old rats. Scale bar 250 µm. The nuclei have been stained with DAPI (blue).

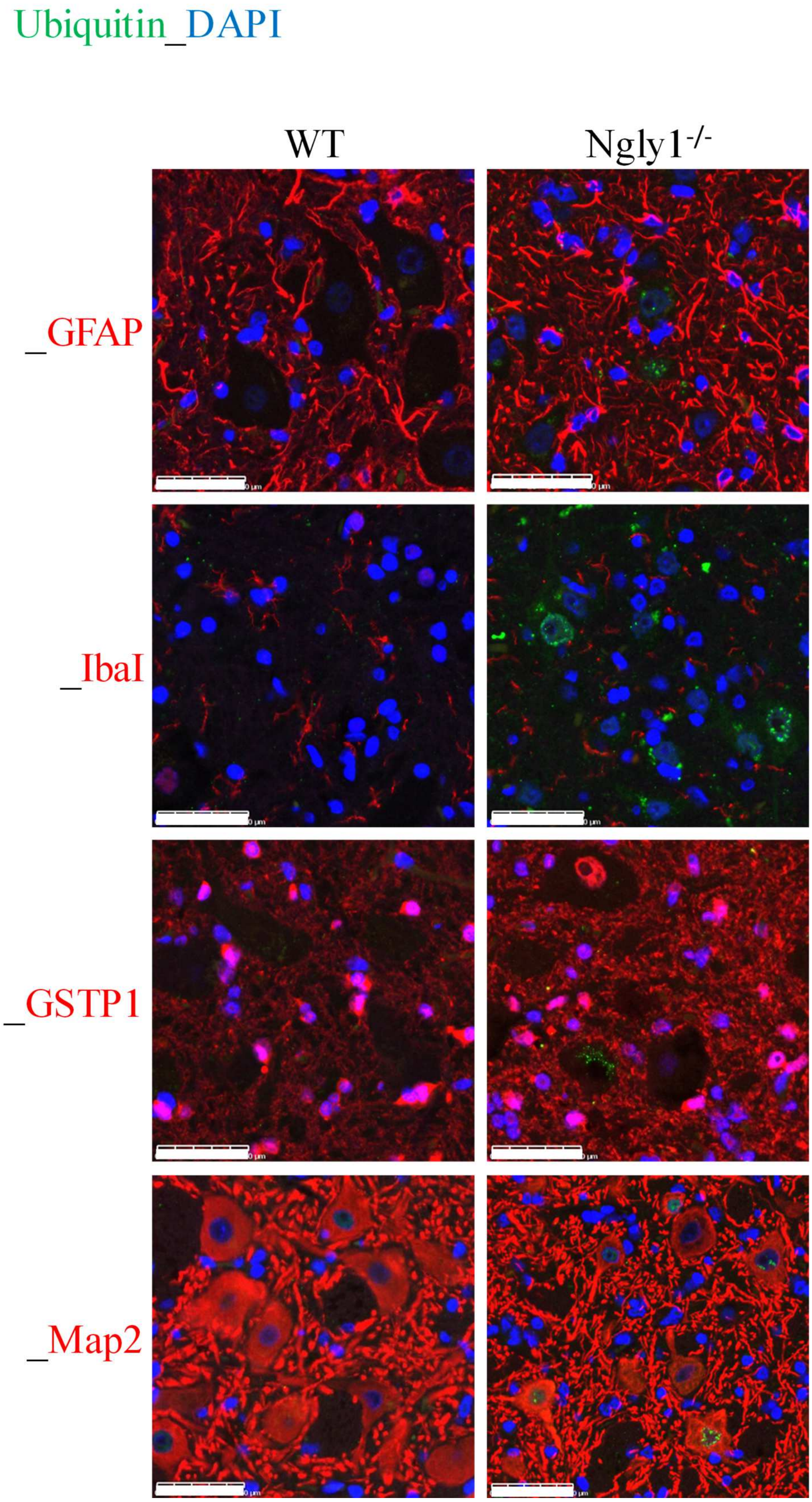

Supplementary Figure 9

Immunohistochemical staining with ubiquitin (green) and glial or neuronal markers (red) in the brains of 29-week-old rats, using antibodies to ubiquitin, GFAP (a marker for astrocytes), IbaI (a marker for microglia), GSTP1 (a marker for oligodendrocytes), and Map2 (a marker for mature neurons). The majority of the cells with ubiquitin-positive proteins were positive for a mature neuronal marker, Map2. The nuclei have been stained with DAPI (blue). Scale bar 50 µm.

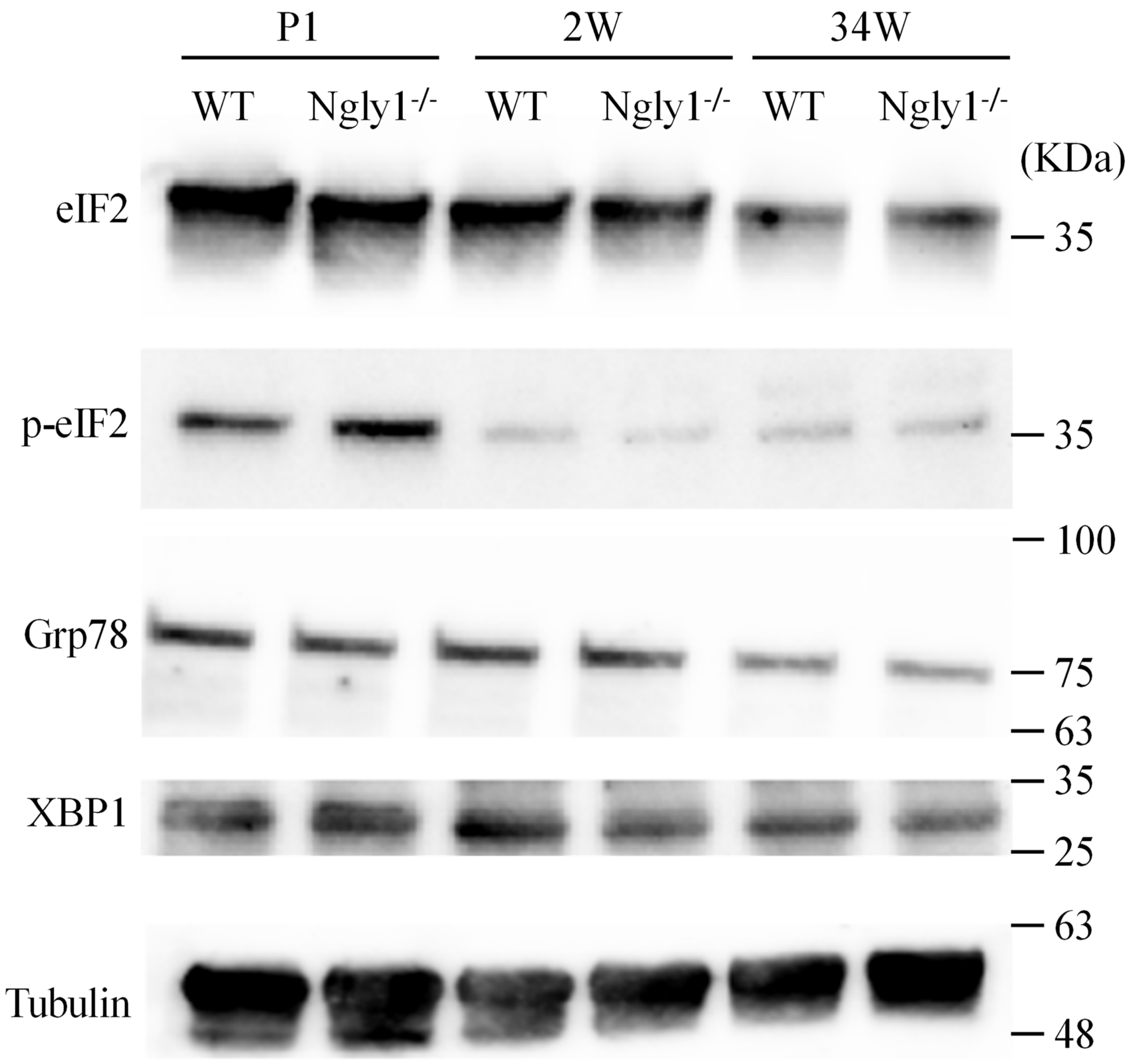

Supplementary Figure 10

Endoplasmic reticulum stress markers were not altered in the brains of *Ngly1−/−* rats. Results of immunoblotting using brain lysates. The expressions of ER stress markers, eIF2, p-eIF2, Grp78 and XBP1, were not affected by loss of rat *Ngly1* at the ages of postnatal day 1 (P1), 2 weeks (2W), and 34 weeks (34W). α-Tubulin was used as a loading control.

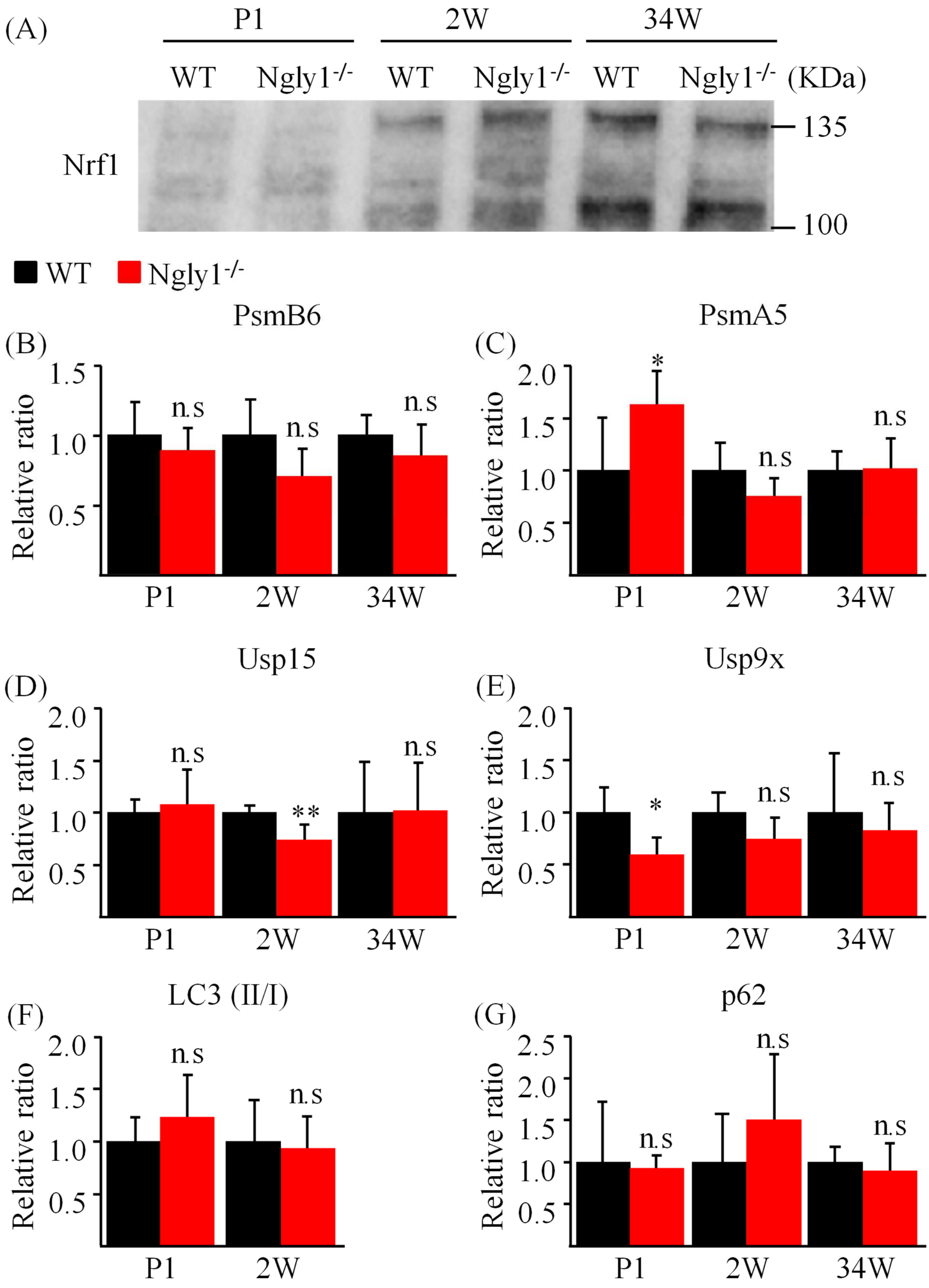

Supplementary Figure 11

No clear change in the level of expression of NRF1-regulating genes and autophagy markers in the brain. Results of immunoblotting using brain lysates of WT or *Ngly1*−/− rats at the ages of postnatal day 1 (P1), 2 weeks (2W), and 34 weeks (34W). (A) NRF1 expression was not altered between WT and *Ngly1*−/− rats. (B–G) The expressions of NRF1-regulating genes, PSMB6 (B), PSMA5 (C), USP15 (D), and USP9X (E), and autophagy markers, LC3 (F) and p62 (G) indicated no clear difference between WT and *Ngly1*−/− rats. α-Tubulin was used as a loading control.

Supplementary Video 1

Abnormal hindlimb clasping of *Ngly1*−/− rats, when suspended by the tail.

Supplementary Video 2

WT rats splaying their hind limbs away from the trunk of their body when suspended by the tail.

Supplementary Video 3

Gait abnormality of 29-week-old *Ngly1*−/− rats.
